# Maternal Dietary Fiber Intake During Lactation and Human Milk Oligosaccharide Fucosylation: a PRIMA Birth Cohort Study

**DOI:** 10.1002/mnfr.70165

**Published:** 2025-06-30

**Authors:** Anneke H. Hellinga, Samanta Cajic, Hanneke F. Linde, Arthur H. van Stigt, Jeanne H. M. de Vries, Elske M. Brouwer‐Brolsma, René Hennig, Erdmann Rapp, Marko Mank, Bernd Stahl, Aletta D. Kraneveld, Jeanette H. W. Leusen, Louis Bont, Belinda van't Land, the PRIMA group

**Affiliations:** ^1^ Center for Translational Immunology University Medical Center Utrecht Utrecht the Netherlands; ^2^ glyXera GmbH Magdeburg Germany; ^3^ Division of Human Nutrition and Health Wageningen University & Research Wageningen the Netherlands; ^4^ Max Planck Institute for Dynamics of Complex Technical Systems Magdeburg Germany; ^5^ Danone Research & Innovation Utrecht the Netherlands; ^6^ Department of Chemical Biology and Drug Discovery Utrecht Institute for Pharmaceutical Sciences (UIPS) Utrecht University Utrecht the Netherlands; ^7^ Department of Neuroscience Faculty of Science VU University Amsterdam the Netherlands; ^8^ Department of Pharmacology Utrecht Institute for Pharmaceutical Sciences (UIPS) Faculty of Science Utrecht University Utrecht the Netherlands; ^9^ Department of Paediatric Immunology and Infectious Diseases Wilhelmina Children's Hospital/University Medical Center Utrecht Utrecht the Netherlands; ^10^ ReSViNET foundation Zeist the Netherlands

**Keywords:** dietary fiber, fucosylation, human milk oligosaccharides, maternal diet, secretor status

## Abstract

Human milk oligosaccharides (HMOs) have an important role in the microbiome and immune system development of breastfed infants. Previous explorative studies indicated an association between maternal carbohydrate intake, including dietary fibers, and specific fucosylated HMOs in human milk (HM). Here, we aim to test whether the intake of dietary fibers by breastfeeding mothers is associated with the level of HMO‐bound fucose in HM samples within a prospective birth cohort study. We assessed dietary fiber intake of healthy mothers (*n* = 164). HMO levels were semi‐quantified in HM samples collected at 1 month postpartum. We found no correlation between fiber intake and HMO‐bound fucose levels. However, secretor mothers (*β* = 2.22, *p* < 0.001) and mothers with a baby girl showed a positive correlation (*β* = 0.41, *p* = 0.016) with the level of HMO‐bound fucose. In contrast, vaginal delivery negatively correlated with the level of HMO‐bound fucose (*β* = −4.93, *p* = 0.008). Overall, there was no association between maternal fiber intake and HMO‐bound fucose levels. Delivery mode, secretor status, and infant sex emerged as the dominant factors associated with HMO fucosylation in HM. Future research should investigate mechanisms underlying HMO fucosylation and its relevance for infant's health.

## Graphical abstract

Healthy breastfeeding women were recruited within the prospective birth cohort PRIMA. We collected a fresh human milk sample at 1 month postpartum to assess relative human milk oligosaccharide (HMO), HMO‐bound fucose levels, and secretor status. In univariate and multivariate linear regression analysis, we did not observe an association between habitual fiber intake and HMO‐bound fucose levels (both regarded prebiotic in nature) within human milk. *Created with BioRender.com*.

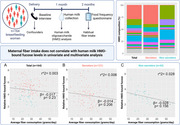

**Abbreviations**
2'‐FL: 2'‐fucosyllactose
3‐FL: 3‐fucosyllactose
3'‐SL: 3'‐sialyllactose
6'‐SL: 6'‐sialyllactose
β4’‐GL: β1‐4‐galactosyllactose
β6’‐GL: β1‐6‐galactosyllactose
DFL: difucosyllactose
DSLNT: disialyllacto‐N‐tetraose
FUT: fucosyltransferase
GlcNAc: *N*‐acetyl‐glucosamine
HM: human milk
HMO: human milk oligosaccharide
IS: internal standard
*Le+*
: Lewis‐positive
*Le−*
: Lewis‐negative
LNDFH I/II: lacto‐N‐difucohexaose I/II
LNFP I/II/III/V: lacto‐N‐fucopentaose I/II/III/V
LNH: lacto‐N‐hexaose
LNnH: lacto‐N‐neohexaose
LNT: lacto‐N‐tetraose
LNnT: lacto‐N‐neotetraose
LSTa/b/c: sialyllacto‐N‐tetraose a/b/c
MFLNH I/II/III: monofucosyllacto‐N‐hexaose
*Se+*
: secretor
*Se−*
: non‐secretor

## 1 Introduction

Human milk oligosaccharides (HMOs) constitute the third most abundant fraction of biomolecules in human milk (HM) next to lactose and lipids. HMOs contribute to the healthy development of neonates and infants in various ways [1, 2]. HMOs are, for example, not digested by the infant but are mostly degraded by the infant's colonic microbiome, thereby serving an important role in the development of the infant's intestinal microbiome. Additionally, HMOs play a role in the development of infant's immune system by direct interaction with local immune cells in the gastrointestinal tract and other sites [3, 4, 5].

HMOs are lactose derivatives, enzymatically elongated with fucose, galactose, *N‐*acetyl‐glucosamine (GlcNAc), and sialic acid, resulting in over 160 different known structures [6, 7, 8]. The pivotal factor of variation in HMO composition and fucosylation is maternal genetics. Polymorphisms in the fucosyltransferase (FUT) (*Se*) and FUT3 (*Le*) genes affect fucosylation via α1,2‐ or α1,4/1,3‐linkages, respectively, classifying individuals into four milk groups based on Secretor (*Se+* and *Se−*) and Lewis (*Le+* and *Le−*) status [9, 10, 11]. Milk group I (*Se+/Le+*) is characterized by high concentrations of fucosylated HMOs, particularly 2’‐FL, with fucose groups via both linkages. Mothers from milk group II (*Se−/Le+*) only produce fucosylated HMOs with fucose bound via α1,2‐linkages, whereas mothers from milk group III (*Se+/Le−*) produce only fucosylated HMOs with fucose linked via an α1,4/1,3‐linkage. Milk group IV (*Se−/Le−*) is characterized by very low levels of fucosylated HMOs in the milk [12].

While there is consensus on the relevance of maternal FUT2 and FUT3 genes, the origin of the remaining HMO variation remains debated [13]. For polyunsaturated fatty acids, the maternal dietary intake has been associated with their respective levels in human milk [14]. Maternal diet has also been suggested as a determinant of HMO composition. The Canadian study by Azad et al. found no associations between maternal diet and HMO composition after adjusting for multiple comparisons [13]. Similarly, HMO levels remained stable over the course of 6 h after a meal with a sugar‐sweetened beverage [15]. Contrastingly, the level of HMO‐bound fucose was increased after an 8‐day intervention with a galactose‐rich diet, versus glucose‐rich, in a cross‐over design [16]. In addition, a small study with 12 secretor participants found a positive correlation between intake of carbohydrates and fruit with relative levels of HMO‐bound galactose and fucose [17]. A Spanish‐Mediterranean cohort observed that maternal diet was differently associated with the HMO composition of secretors and non‐secretors. For secretors, they found that intake of dietary fibers and (poly)phenols was negatively associated with total HMOs, but positively associated with several individual fucosylated HMOs [18]. While several studies show diet‐associated HMO variations, data are yet too limited to conclude on specific diet‐HMO comparisons.

Based on the preliminary findings of the first explorative studies, we hypothesize that the intake of dietary fiber during lactation is positively associated with the level of HMO‐bound fucose in human milk. Here, we present the results in a relatively large observational study with extensive data on participant and sample characteristics, allowing multivariate analysis. Consensus on the relevance of maternal diet for HMO composition will allow tailoring dietary advice for lactating women in order to optimize infant's health and development.

## 2 Experimental Section

### 2.1 Participants and Data Collection

We recruited participants from the obstetric wards of two hospitals in Utrecht, the Netherlands, as part of the prospective PRIMA birth cohort. The study was approved by the Medical Research Ethics Committee Utrecht (NL74946.041.20), and all parents provided written consent for participation [19]. We contacted generally healthy mother‐infant pairs during the first week postpartum, if the mother initiated breastfeeding and intended to continue breastfeeding beyond 3 months postpartum. For this study, only participants with complete data from the food frequency questionnaire and HMO composition of a HM sample at 1 month postpartum were included (*n* = 164).

We collected baseline data via telephone interview. Baseline information included age, pre‐pregnancy weight, height, and educational level of the mother, as well as the infant's sex, birthweight, gestational age, delivery mode, and number of siblings. At 1 month postpartum, participants pumped before breakfast and stored the sample in the refrigerator. We collected fresh HM samples of at least 5 mL and processed these within 24 h to extract the water fraction, which was stored at −80°C until HMO analysis. To assess short‐term fiber intake during the 24 h before HM sampling, participants completed a 24 h dietary recall on the morning of collection using the web‐based Compl‐eat tool [20]. Trained researchers reviewed the recalls and contacted participants by phone if clarification was needed. At 2 months postpartum, participants completed a validated semi‐quantitative 183‐food item food frequency questionnaire (FFQ) to assess energy and dietary fiber intake from the previous month as a measure of long‐term or habitual intake [21, 22]. Adherence to the Dutch dietary guidelines was assessed using the Dutch Healthy Diet‐15 Index (DHDI), which excluded salt and coffee components, resulting in a maximum score of 130 [23]. Energy and fiber intake was estimated from the 24 h recall and FFQ based on the consumption frequency, portion size, and nutrient content as indicated in the Dutch food composition table [22].

Additional information for methods on baseline information and sample collection are in the Supporting Information.

### 2.2 Human Milk Oligosaccharide Sample Preparation, Measurement, and Data Analysis

HMO composition and levels of all samples were analyzed based on a method previously published [24]. We prepared samples for analysis using glyXprep sample preparation kit (glyXera GmbH, Germany), modified for qualitative and quantitative high‐throughput HMO analysis. Briefly, an internal standard (IS) with known concentration was spiked to each sample prior to analysis, to semi‐quantify peak levels between samples. After denaturation, HMOs and IS were fluorescently labeled and purified, before samples were measured. HMO analysis was performed using glyXboxCE (glyXera GmbH, Germany), based on capillary gel electrophoresis coupled with laser‐induced fluorescence detection (xCGE‐LIF). Data processing and evaluation were adjusted and performed using a tailored version of the glyXtoolCE software (glyXera GmbH, Germany), identifying 135 peaks. %Total Peak Height (TPH) of a peak was calculated relative to the TPH of the entire same sample. %TPH were normalized to IS (%IS) to semi‐quantify and compare between samples. In addition, %Total Peak Area (TPA) was calculated and strongly correlated with %TPH (Figure S1), leading to similar results of the analysis (data not shown).

We semi‐quantified HMO‐bound fucose for 21 selected HMO peaks, for which the structure is known. By summing %IS for detected peaks assigned to monofucosylated and difucosylated HMOs, multiplied by the number of fucose groups within the annotated HMOs, the total HMO‐bound fucose level was assessed. Overlapping peaks of two HMOs were handled as one in the data analysis and presentation (e.g., 2’‐FL# containes traces of β4’‐GL). Participants were classified as secretors based on the presence of secretor‐specific HMOs (like 2’‐FL, DFL, LNFP I) in their HM sample [10, 24].

Additional information for methods of HMO analysis is in the Supporting Information.

### 2.3 Statistical Analysis

The primary aim was to test for an association between dietary fiber intake and %IS fucose. This was tested with univariate and multivariate linear regression models. For the latter, we identified potential confounders through literature research and expert knowledge (Figure S1). Variables were initially included in the multivariate model if *r* > 0.7 for variable compared to %IS fucose. If the correlation of two independent variables was *r* > 0.8 and/or biologically irrelevant to include both (e.g., BMI and weight), the variable with the least correlation was not included in the model. As a rule of thumb, we considered no more than one independent variable for every 10 participants in the model to reduce the risk of an overfitting model. If the selection of independent variables exceeded this, the variables with the lowest absolute correlation coefficient were not included. Then, we applied backwards selection to identify the best‐fitted model. We assessed model assumptions as not violated.

The difference in self‐reported maternal energy and fiber intake between secretors and non‐secretors was tested by the Wilcoxon rank sum test, as both the dietary and HMO data concern ranked (semi‐quantitative) data. Similarly, the difference between the level of individual HMOs between secretors and non‐secretors was tested by Wilcoxon rank sum test and adjusted for multiple testing by the false discovery rate method. The correlation between fiber consumption and %IS of each of the fucosylated HMOs was tested with the Pearson correlation.

All statistical analyses were performed using Rstudio (v2023.06.0 Build 421) using the “factoextra,” “lm,” “ggcorplot,” and “ggplot2” packages.

## 3 Results

### 3.1 Participants and Dietary Intake

We included 164 participants with complete data on habitual fiber intake and HMO levels (Table 1). Most women had received higher education and had a healthy BMI before pregnancy and at 2 months postpartum. The majority of infants were born via vaginal delivery with a healthy birthweight; five infants had a low birthweight ranging from 2000 to 2500 g. Exclusively breastfeeding was observed in 95% of participants at the time of sample collection. Based on the HMO profile, 121 women were classified as secretors (73.8%) and 43 as non‐secretors (26.2%).

**TABLE 1:** Baseline characteristics for *N* = 164 participating mother‐infant dyads.

		*N*	Mean ± SD or percentage (%)
**Mother**			
Age, y			33.9 ± 3.6
Pre‐pregnancy BMI, kg/m^2^			23 ± 3.5
	Missing	1	0.6%
BMI at 2 months, kg/m^2^			24.1 ± 3.4
	Missing	10	6.1%
Educational level mother			
	Lower	0	0.0%
	Middle	15	9.1%
	Higher	149	90.9%
**Infant**			
Sex	Male	76	46.3%
Birth weight, g			3454 ± 543
Mean gestational age, weeks			39.6 ± 1.6
Delivery			
	Vaginal	115	70.1%
	C‐section	49	29.9%
Siblings			
	0	87	53.0%
	1	50	30.5%
	2	25	15.2%
	3	2	1.2%
**Breastfeeding and sample**			
Lactation stage (days postpartum)			26.2 ± 6.3
Proportion breastfeeding, %			95.1 ± 17
	Missing	*5*	3.0%
Season during collection			
	Spring (March–May)	34	20.7%
	Summer (June–August)	53	32.3%
	Fall (September–November)	43	26.2%
	Winter (December–February)	34	20.7%
HMO group			
	I (Se+, Le+)	106	64.6%
	II (Se−,Le+)	39	23.8%
	III (Se+, Le−)	15	9.1%
	IV (Se−, Le−)	4	2.4%
Type of HM sample			
	Foremilk	23	14.0%
	Hindmilk	35	21.3%
	Fore‐ and hindmilk	14	8.5%
	Full expression (1 or 2 breasts)	92	56.1%

Table 2 shows that fiber consumption during the second month postpartum (FFQ) and the day before sampling (24 h recall) was similar but slightly lower than recommended for the general Dutch adult population [25]. Long‐ and short‐term assessments of dietary fiber intake were not significantly different between secretor and non‐secretor mothers. The average intake of fiber remained relatively stable with no significant difference in energy‐adjusted fiber intake FFQ assessments at 2 and 4 months postpartum (data not shown). Average energy intake during the second month postpartum and the day before sampling was within the range of average energy requirement for Dutch women [26] and did not significantly differ between secretors and non‐secretors. Adherence to the Dutch dietary guidelines, as evaluated by the Dutch Healthy Diet Index, also showed no significant difference between secretors and non‐secretors.

**TABLE 2:** Self‐reported maternal energy and fiber assessed intake (*n* = 164).

	Total		Non‐secretors		Secretors		*p*	(Reference)
	*N*	Mean ± SD	*n*	Mean ± SD	*n*	Mean ± SD		
**FFQ at 2 months postpartum**	164		43		121			
Energy, avg kcal/day		2231 ± 652		2282 ± 739		2213 ± 6215	0.79	(2020–2880 kcal/day^a^)
Dietary fiber, avg g/day		26.4 ± 8.9		26.7 ± 8.5		26.2 ± 9.1	0.78	(14 g/1000 kcal^b^)
Dutch Healthy Diet Index		85.1 ± 14.6		83.0 ± 15.3		85.9 ± 14.3	0.25	(Max. score 130)
**24 h dietary recall at 1 month postpartum**	163		43		120			
Energy, kcal		2350.3 ± 665.7		2368.1 ± 682.1		2343.9 ± 662.5	0.66	
Dietary fiber, g		27.5 ± 12.1		27.1 ± 16		27.6 ± 10.5	0.27	

*Note*: Energy and fiber intake are presented with mean ± standard deviation (SD). The difference in intake between secretors and non‐secretors was tested by the Wilcoxon rank sum test and probability is presented.

Abbreviations: avg, average; FFQ, food frequency questionnaire; *p*, probability.

^a^ Average energy requirement is dependent on physical activity level [26].

^b^ Guideline for dietary fiber intake for the general Dutch population [25].

### 3.2 HMO Abundance and Composition Differ Between Secretors and Non‐Secretors

Principal component analysis of the complete cohort clearly shows that samples from secretors clustered separately from those of non‐secretors (Figure S2). Annotated LNFP V, 2’‐FL, LNFP II, 3‐FL, LNDFH II, LNFP I were among the 10 main contributors of the variation explained by principal component (PC) 1, in decreasing order of contribution. MFLNH II+III was the only annotated HMO among the 10 main contributors to PC2 (data not shown).

Participants were distinguished as secretors based on the presence of 2’‐FL, DFL, LNFP I, and LNDFH I in their HM samples. Notably, LNnH+LNH, LNnT, LSTc, and MFLNH I were observed to be significantly higher in samples from secretor compared to non‐secretor's (Figure S3). The level of both fucosylated as well as total HMOs was significantly higher in samples of secretors compared to non‐secretors. Conversely, several HMOs were significantly more abundant in non‐secretors samples.

These differences in HMO composition between secretors and non‐secretors were also evident in Figure S4, which shows the mean %TPH (not normalized to IS) of HMOs within the total population and separated by secretor status.

### 3.3 Maternal Fiber Consumption Is Not Associated With HMO Fucosylation

**FIGURE 1:**
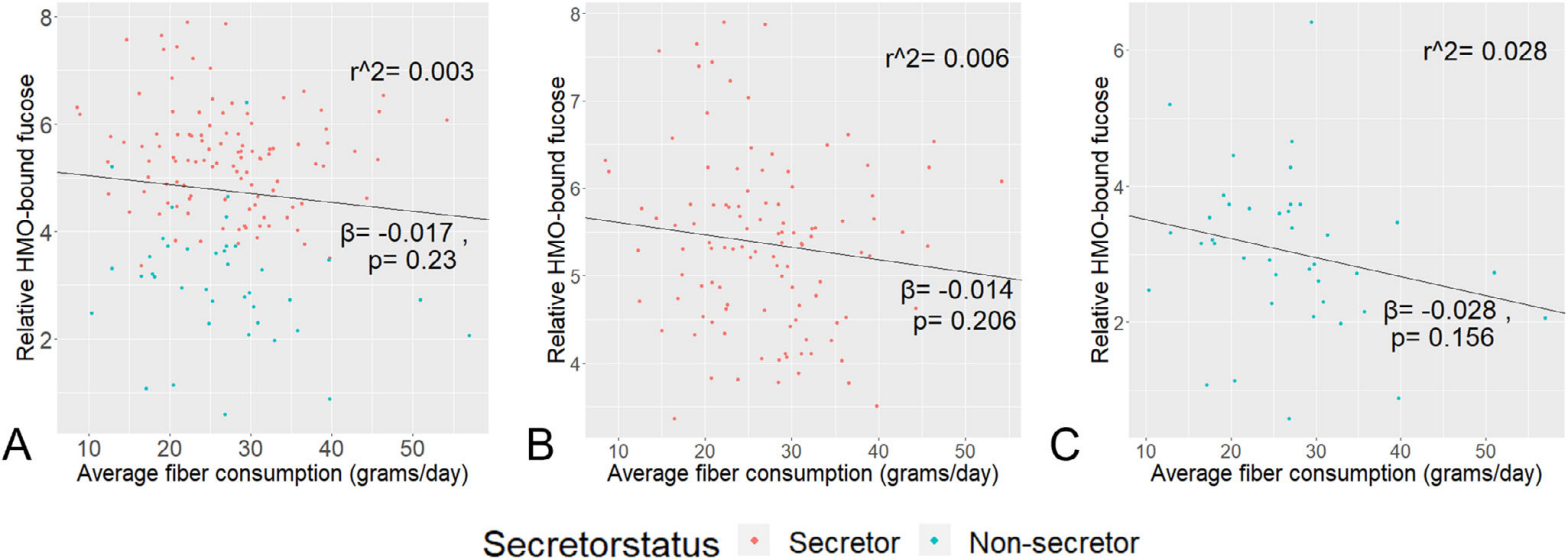
Correlation of fiber consumption of breastfeeding mothers with relative fucosylation of annotated human milk oligosaccharides (HMOs) for total *n* = 164 participants **(A)**, and separately for *n* = 121 secretors **(B)**, and *n* = 43 non‐secretors **(C)**. Regression lines and coefficients from the crude models are plotted for average fiber consumption (g/day) (Table 1). Fiber consumption was assessed by self‐reported intake using a food frequency questionnaire at 2 months postpartum, covering the previous 4 weeks, resulting in an average intake of gram/day. Human milk samples at 1 month postpartum were analyzed for HMOs with multiplexed capillary gel electrophoresis with laser‐induced fluorescence detection. The absolute peak height of annotated fucosylated HMOs (2’‐FL, 3‐FL, DFL, LNDFH I and II, LNFP I, II, III, V, and MFLNH I, II, and III) are divided by absolute peak height of the internal standard (IS) to calculate %IS. Fucosylation is assessed by multiplying %IS of HMO by the amount of fucose groups within the HMOs, summed up for all annotated HMOs per sample.

We found that habitual fiber consumption was not associated with relative HMO‐bound fucose levels in either the complete sub‐study or when participants were stratified by secretor status in our univariate models (Figure 1). This finding was confirmed in our multivariate model for the total population (Table 3).

**TABLE 3:** Multiple linear regression analysis results for crude and adjusted models for habitual dietary fiber intake on relative HMO‐bound fucose for total and subsets of study population.

Model	Model fit	Variable	*β*	*p*
Total population (*n* = 164)				
Crude model	*R* ^2^ = 0.003; se = 1.448	Average fiber consumption (g/day)	−0.02	0.230
Adjusted model	*R* ^2^ = 0.537; se = 0.987	Average fiber consumption (g/day)	−0.02	0.294
		Secretor status: Secretor	2.33	**<0.001**
		Delivery: vaginal	−4.93	**0.008**
		Energy intake (kcal)	< −0.01	0.895
		Infant's sex: girl	0.41	**0.016**
		Maternal body weight	0.03	0.275
**Secretors (*n* = 121)**				
Crude model	*R* ^2^ = 0.006; se = 0.980	Average fiber consumption (g/day)	−0.01	0.206
Adjusted model	*R* ^2^ = 0.128 se = 0.918	Average fiber consumption (g/day)	0.009	0.661
		2mBMI	0.06	0.085
		Infant's sex: girl	0.41	**0.026**
		Lewis status: positive	−0.59	**0.034**
		Days postpartum	−0.04	**0.009**
		Season: winter (ref: autumn)	−0.65	**0.010**
		Energy intake (kcal)	−0.0002	0.407
		Birthweight	0.0002	0.120
**Non‐secretors (*n* = 43)**				
Crude model	*R* ^2^ = 0.028; se = 1.131	Average fiber consumption (g/day)	−0.03	0.156
Adjusted model	*R* ^2^ = 0.471; se = 0.834	Average fiber consumption (g/day)	−0.05	**0.045**
		Energy intake (kcal)	−0.0002	0.399
		Lewis status: positive	2.4	**<0.001**
		Delivery: vaginal	−0.58	0.06

*Note*: Model parameters for crude and adjusted multiple linear regression are presented. Habitual dietary fiber intake was assessed by a food frequency questionnaire 2 months postpartum. HMO‐bound fucose was semi‐quantified in human milk samples collected at 1 month postpartum.

Abbreviations: *β*, *β*‐coefficient from multiple linear regression model; HMO, human milk oligosaccharide; *p*, probability; se, residual standard error.

However, secretors, and mothers with a baby girl, had significantly higher levels of relative HMO‐bound fucose levels than non‐secretors, and mothers with a baby boy, respectively. Mothers who delivered vaginally had significantly lower relative HMO‐bound fucose levels compared to those who delivered via cesarean section (Table 3).

Including only secretors in the multivariate analysis, also no correlation was found between fiber intake and HMO‐bound fucose levels. In accordance with the model for the total population, secretor mothers with a baby girl had significantly higher levels of HMO‐bound fucose. Secretors with a positive Lewis status (HMO group I), or mothers who donated milk during the winter, had significantly lower HMO‐bound fucose levels than secretors with a negative Lewis status (HMO group III), or those who donated milk during the autumn, respectively. In addition, the number of days postpartum was negatively correlated with the level of HMO‐bound fucose in collected milk samples (Table 3).

Including only non‐secretors in the multivariate analysis showed a borderline significant slightly negative correlation between fiber intake and relative HMO‐bound fucose levels. Unlike secretors, non‐secretors with a positive Lewis status (HMO group II) had higher levels of HMO‐bound fucose than Lewis‐negative non‐secretors (HMO group IV) (Table 3).

It is important to note that the three multivariate models examining habitual fiber intake explained only a limited proportion of the variance (adjusted *r* ^2^ = 0.128–0.537). This indicates that a substantial proportion of variance remained unexplained (Table 3).

Table S1 shows the results of both univariate and multivariate analysis examining the relationship between dietary fiber intake in the 24 h prior to sample collection and relative HMO‐bound fucose. Consistent with the analyses on habitual fiber intake, the univariate analysis did not show a significant correlation. In addition, there was no correlation between fiber intake in the prior 24 h and relative HMO‐bound fucose for both the total population and secretors when analyzed separately. Unlike the findings for habitual fiber intake, there was no correlation between fiber intake in the 24 h prior to sample collection and relative HMO‐bound fucose in non‐secretors.

Figure S5 shows that habitual dietary fiber intake was not significantly correlated with any of the individual fucosylated HMOs, neither for the total sub‐study population nor for secretors and non‐secretors separately.

## 4 Discussion

In this PRIMA sub‐study, we analyzed the relationship between postpartum maternal dietary fiber consumption and HMO fucosylation. Despite observing considerable variation in HMO fucosylation, fiber consumption does not explain (the major part of) the variation. The primary factor observed to contribute to variation was delivery mode, followed by secretor status and infant sex. For secretors, the timing of sample collection, specifically winter or autumn sample collection, was the major contributor to the variation observed. For non‐secretors, Lewis status was the primary driver for HMO fucosylation. These findings underscore the complexity of HMO fucosylation and highlight that while maternal fiber intake may have limited relation, other variables such as delivery method, secretor status, and seasonal effects may be related to HMO fucosylation.

HMOs are produced in the mammary glands through elongation of lactose with glucose, galactose, GlcNAc, fucose, and/or sialic acid [7, 8, 27, 28]. This process is catalyzed by a variety of glycosyltransferases, with FUT2 being most well‐known for facilitating fucosylation via an α1,2‐linkage [9, 10]. However, the major part of the complex process of HMO biosynthesis remains poorly understood and different approaches, including network‐based modeling, are taken to unravel the reactions and enzymes involved [7, 29]. Still, it has been shown that orally consumed [13] C‐galactose is incorporated into milk composites [30], though no association has been observed between consumption of lactose and HM levels of lactose [14, 31, 32, 33]. In addition, maternal diet and specifically dietary fiber could potentially influence the expression of genes encoding the numerous enzymes involved in HMO biosynthesis [34]. Dietary fibers are fermented by the gut microbiome to short‐chain fatty acids (SCFAs), such as acetate, butyrate, and propionate. SCFAs are known to modulate histone acetyltransferases and histone deacetylases, thereby influencing gene expression. This is further substantiated by the observation that SCFAs upregulated FUT2 expression in epithelial cells [35]. However, further mechanistic or intervention studies (with for instance labeled [13]C‐atoms) are necessary to elucidate on if, and how, dietary fibers and other dietary components could have a role in the metabolic pathway of endogenous HMO synthesis.

In recent years, explorative observational as well as some intervention studies have investigated the influence and association of maternal diet with HMO composition. While some indicated a positive correlation between dietary fiber intake and HMO‐bound fucose or levels of fucosylated HMOs [17, 18, 36], we did not observe this in our study. Quin et al. observed a significantly positive correlation between fiber intake during the 24 h prior to sample collection and HMO‐bound fucose in HM samples collected at 5 months postpartum from 12 secretor mothers [17]. However, we found no correlation between fiber intake in the 24 h prior to sample collection and the level of relative HMO‐bound fucose in the total sub‐study population, secretors nor non‐secretors. Similarly, findings from other studies linking maternal diet to various individual fucosylated HMOs were not replicated here [18, 36, 37, 38]. Differences in study population, study design, including sample size, or lactation stage may explain the discrepancy.

One study population characteristic that might explain the inconsistency is the nutritional status of breastfeeding mothers. A study that followed 94 women during the first 6 months postpartum found that 34.8% were undernourished. In this study population, the household hunger scale index was inversely correlated with the levels of 2’‐FL and 3‐FL, and LNDFH I was less abundant in the milk of mothers from mildly food insecure households compared to those from food secure households. Remarkably, HMO building block lactose was significantly higher in HM of mothers from moderately food insecure households compared to food secure households, while the lactose levels were negatively associated with meat and fish consumption [38]. Although this and other observational studies do not seem to include undernourished participants, as suggested by a Healthy Eating Index, reference values and/or body mass index, it might still be that part of the participants have inadequate dietary fiber intake.

In the context of maternal fiber consumption, we observed that way of delivery, maternal secretor status, and infant sex significantly associated with relative HMO‐bound fucose levels. The relevance of maternal Secretor and Lewis status for HMO composition is well established. Polymorphisms in the maternal *FUT2 and FUT3* genes determine the ability to synthesize specific HMOs through α1,2‐ and α1,3‐/α1,4‐fucosylation, respectively, thereby influencing the overall HMO composition [11]. Similarly, HMO composition is observed to change over lactation time and vary over the globe, as is the abundance of secretors [39, 40, 41]. We aimed to primarily study the association between maternal dietary fiber intake and HMO‐bound fucose levels. In this context, delivery mode, maternal secretor status, and infant sex significantly associated with relative HMO‐bound fucose levels. The relevance of delivery mode was previously recognized, with vaginal delivery associated with higher levels of relative HMO‐bound fucose or specific fucosylated HMOs than cesarean section [42]. Interestingly, in the context of habitual fiber intake, we observed lower levels of relative HMO‐bound fucose in milk of mothers who delivered vaginally. Other studies did not observe a significant association between delivery mode and HMO‐bound fucose [13, 43, 44]. The effect of delivery is thus not consistently observed in different studies. Similarly, we did not observe a significant difference in relative HMO‐bound fucose levels between vaginal delivery and cesarean section for the total population, or secretors and non‐secretors separately (data not shown). It may be that an effect of delivery is temporary and a result of changes in (stress) hormones, physiological stress, and/or antibiotic use [40, 45, 46]. Together, this again advocates for further studies on the mechanisms and factors influencing HMO composition in human milk.

Though the relevance of HMOs on an infant's microbiome and immune system development has become clearer over the past decade [2, 47], it is not fully clear to what extent fucosylation is essential in this. It is observed that stool from infants fed with secretor milk has a higher abundance of bifidobacteria than infants fed with non‐secretor milk [2, 48]. In addition, while HMOs in general have (in)direct anti‐pathogenic effects [2], only fucosylated HMOs act as decoy receptors for the respiratory syncytial virus and norovirus [49]. Only for the binding interaction with norovirus, it is known that the binding is specifically via the fucose groups of 2'‐FL and 3‐FL [49, 50]. In a large Canadian study it was observed that for high‐risk breastfed infants, the level of certain fucosylated HMOs was inversely associated with the prevalence of recurrent wheeze [11]. This indicates that the level of fucosylated HMOs in HM might be relevant for the microbiome development and health of breastfed infants and should therefore be studied further.

This relatively large study examined a hypothesis‐driven association between dietary fiber intake and HMO fucosylation, reducing the risk of chance finding. Other strengths include dietary assessment during lactation, assessing both long‐term and short‐term fiber intake, relatively standardized HM sampling, and elaborate information on sampling methods and baseline characteristics, allowing for relevant adjustments. Limitations include assessing long‐term dietary fiber intake based only on the month after HM sampling and semi‐quantitative (as opposed to quantitative) data on HMO abundance, and the inability to distinguish between soluble and insoluble fibers. The (natural) underrepresentation of participants from HMO group IV (*Se−/Le−*) could limit in the analysis. Other limitations are the homogeneous population with a non‐representative high proportion of participants with a high education level, affecting generalizability, and limited effect size due to limited variation in fiber consumption.

With a reasonably large study population and targeted analysis, our study did not observe a correlation between maternal fiber consumption and HMO‐bound fucose. Observational longitudinal cohorts in diverse populations, also considering maternal nutritional status, as well as randomized controlled trials, are needed to conclude on this. Even more, the potential impact of possibly altered HMO fucosylation on infant's health should be taken into account.

## Conflicts of Interest

B.L., M.M., and B.S. are (partly) employed by Danone Research & Innovation (Utrecht, the Netherlands). E.R. is founder and CEO of glyXera GmbH (Magdeburg, Germany). R.H. is co‐founder and CSO of glyXera. S.C. is employee of glyXera. E.B.B. received funding from Ausnutria BV. None of the authors declared further conflicts of interest.

## Supporting information




**Supporting File 1**: mnfr70165‐sup‐0001‐SuppMat.docx.
[Correction added on 2 July 2025, after first online publication: Supporting information has been updated]

## Data Availability

Data available on request from the authors
