# Supplementary material for: Maternal Dietary Fiber Intake During Lactation and Human Milk Oligosaccharide Fucosylation: a PRIMA Birth Cohort Study
Source: Mol Nutr Food Res. 2025 Jun 30;69(20):e70165. doi: 10.1002/mnfr.70165 (PMC12538538; doi:10.1002/mnfr.70165)
Supplement: Supplementary file 1 — Supporting File 1: mnfr70165‐sup‐0001‐SuppMat.docx. [Correction added on 2 July 2025, after first online publication: Supporting information has been updated] [file MNFR-69-e70165-s001.docx]

**Supplementary materials**

Supplemental methods

Supplemental table 1. Results of multiple linear regression analyses, results for crude and adjusted models with short term fiber intake for total and subsets of study population

Supplemental figure 1. Correlation matrix (ggcorrplot) for selection of independent variables for multiple linear regression model for total study population (n=164), secretors (n=121) and non-secretors (n=43).

Supplemental figure 2. Principal component analysis (PCA) of %IS of all detected HMOs. Scatters and ellipses are drawn based on predefined secretorstatus.

Supplemental figure 3. Levels of major human milk oligosaccharides, fucosylation and total HMOs for non-secretors (n=43) and secretors (n=121).

Supplemental figure 4. Proportion of human milk oligosaccharides separated for non-secretors (n=43), secretors (n=121) and for total population (n=164).

Supplemental figure 5. Correlation of fiber consumption of breastfeeding mothers with IS-normalized peak height of fucosylated HMOs for total sub-study population (n=164), and separately for secretors (n=121) and non-secretors (n=43).

**Supplemental methods**

*Details on baseline and sample collection*

Education level was categorized as low when no education was completed or, lower secondary or intermediate vocational education was completed, as middle when intermediate vocational education was achieved, as higher when higher vocational education, university or post-academic degree was completed by the participant. Breastfeeding exclusiveness was assessed by the type of feeding (human milk and/or formula) and proportion by the parent through the parent-reported bi-weekly questionnaire covering the sample moment. In case the questionnaire was missing (n=16), the average of reported proportions in adjacent questionnaires was used if available (n=11).

Preferably a sample from a full HM expression was collected, but when not preferred by the parent(s) or not feasible, fore- and/or hindmilk was collected. Although arbitrary, foremilk was defined as the first 30ml or 3 minutes pumping, after which it was defined as hindmilk, and reported by the parent(s) during collection. Samples were transported on ice and processed the same day. Samples were centrifuged twice at 600xg to collect the water fraction, which was aliquoted and stored at -80⁰C, within 24 hours after collection. ^[1]^

*Human milk oligosaccharide (HMO) analysis*

HMO analysis was performed via multiplexed capillary gel electrophoresis coupled with laser-induced fluorescence detection (xCGE-LIF; the glyXboxCE™ system of glyXera GmbH, Germany). Samples were prepared for xCGE-LIF analysis using the sample preparation kit glyXprep™ (glyXera GmbH, Germany), modified as follows: Internal standard (IS) with known concentration was spiked to HM water fractions after which the samples were denatured by adding sodium dodecyl sulfate (SDS) and heat treatment (60°C for 10 min). Spiked and denatured HMO samples, were labeled with 8-aminopyrene-1,3,6-trisulfonic acid (APTS) via reductive amination. Afterwards, labeled samples were purified by HILIC-SPE and diluted (1:10 and 1:200). Lastly, a dedicated internal migration time alignment standard was added to each sample.

Data processing and profiling was performed using glyXtool^TM^ software (v0.8.19, glyXera GmbH, Germany). With the tailored software, peak-picking, -integration, and migration time alignment to the internal standard - converting HMO-electropherograms into aligned HMO-fingerprints - was performed, and data-analyses of all samples were manually curated, supported by the software. Limit of quantification (LOQ) was determined using signal-to-noise ratio of each HMO fingerprint. ^[2]^ Values <LOQ were imputed with LOQ of the peak divided by the square-root of 2. Values below limit of detection were omitted in data analysis. A peak was only included if its height was quantified in over 80% of the samples. Peak heights and areas of quantifiable peaks (> LOQ) were added together per sample, resulting in a total peak height (TPH) and total peak area (TPA) (=100%).

[1] A. H. van Stigt, K. Oude Rengerink, K. W. M. Bloemenkamp, W. de Waal, S. M. P. J. Prevaes, T. M. Le, F. van Wijk, M. Nederend, A. H. Hellinga, C. S. Lammers, G. den Hartog, M. J. C. van Herwijnen, J. Garssen, L. M. J. Knippels, L. M. Verhagen, C. G. M. de Theije, A. Lopez-Rincon, J. H. W. Leusen, B. van’t Land, L. Bont, A. C. Knulst, C. K. van der Ent, D. van Baarle, M. H. M. Wauben, N. Y. Rots, E. A. M. Sanders, M. J. N. L. Benders, L. A. M. P. Meulenbroek, B. Stahl, A. D. Kraneveld, B. J. M. Buiteman, T. Voogt, B. van der Meij, B. Lerkvaleekul, E. Voogd, T. A. Lalmahomed, I. M. Brus, D. M. M. van Meerwijk, S. I. E. Jepma, *BMC Infect Dis* **2022**, *22*, DOI 10.1186/s12879-022-07107-w.

[2] S. Ullsten, R. Danielsson, D. Bäckström, P. Sjöberg, J. Bergquist, *J Chromatogr A* **2006**, *1117*, 87–93.

| **Table S1.** Result of multiple linear regression analyses, results for crude and adjusted models with short term fiber intake for the total and subsets of study population | | | | |
| --- | --- | --- | --- | --- |
| **Model** | **Model fit** | **Variable** | **β** | **p** |
| **Total population (n=164)** |  |  |  |  |
| Crude model | R^2^= -0.006; se= 1.457 | Fiber consumption on prior 24h (g) | 0.002 | 0.806 |
| Adjusted model | R^2^= 0.524; se= 1.002 | Fiber consumption on prior 24h (g) | -0.004 | 0.674 |
|  |  | Secretorstatus: Secretor | 2.32 | **<0.001** |
|  |  | Infant’s sex: Girl | 0.40 | **0.022** |
|  |  | Delivery: Vaginal | 0.47 | **0.012** |
|  |  | Energy intake (kcal) | <0.001 | 0.727 |
| **Secretors (n=121)** |  |  |  |  |
| Crude model | R^2^= 0.0007; se= 0.986 | Fiber consumption on prior 24h (g) | 0.01 | 0.301 |
| Adjusted model | R^2^= 0.137; se= 0.916 | Fiber consumption on prior 24h (g) | 0.01 | 0.26 |
|  |  | Lewisstatus: positive | -0.54 | 0.061 |
|  |  | 2mBMI | 0.08 | **0.015** |
|  |  | Infant’s sex: Girl | 0.32 | 0.079 |
|  |  | Lactation days | -0.04 | **0.007** |
|  |  | Season: Winter (ref Autumn) | -0.55 | **0.030** |
|  |  | Season: Summer (ref Autumn) | 0.25 | 0.241 |
|  |  | Energy intake (kcal) | <0.001 | 0.889 |
| **Non-secretors (n=43)** |  |  |  |  |
| Crude model | R^2^= 0.021; se= 1.135 | Fiber consumption on prior 24h (g) | -0.02 | 0.188 |
| Adjusted model | R^2^= 0.478; se= 0.829 | Fiber consumption on prior 24h (g) | -0.01 | 0.184 |
|  |  | Lewisstatus: positive | 2.27 | **<0.001** |
|  |  | Delivery: Vaginal | -0.46 | 0.146 |
|  |  | Energy intake (kcal) | < -0.001 | 0.192 |
| Model parameters for crude and adjusted multiple linear regression (*lm* in Rstudio) are presented. HMO: human milk oligosaccharide, se= residual standard error | | | | |


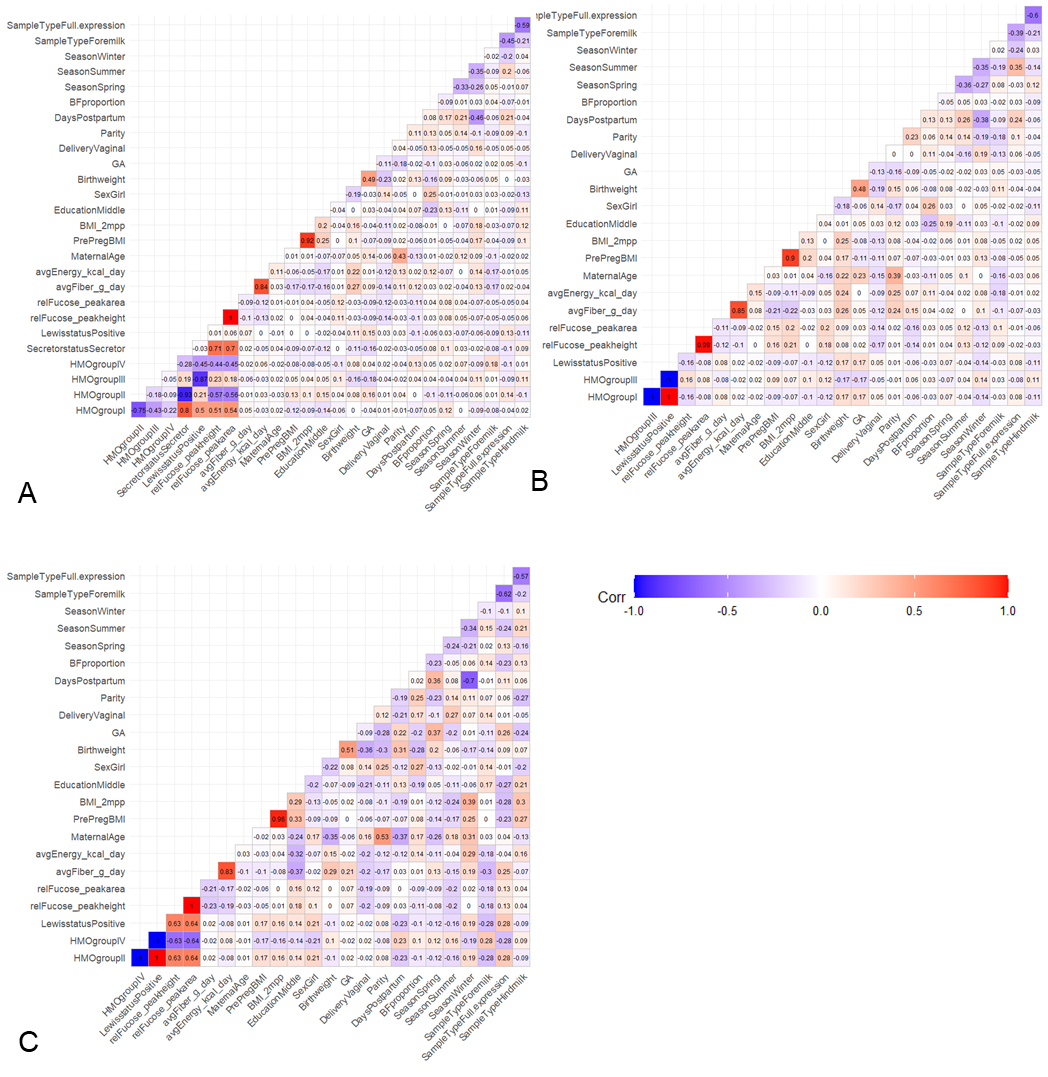


**Supplemental figure 1.** Correlation matrix (*ggcorrplot*) for selection of independent variables for multiple linear regression model on average fiber consumption and relative HMO-bound fucose for A) total study population (n=164), B) secretors (n=121) and C) non-secretors (n=43). Variables were initially included in the multivariate model (***table 1***) if r>0.7 for variable compared to %IS fucose. If correlation of two independent variables was r>0.8 and/or biologically irrelevant to include both (e.g. BMI and weight), the variable with least correlation was not included in the model. As a rule of thumb, no more than one independent variable for every ten participants was considered in the model. If the selection of independent variables exceeded this, the variables with lowest absolute correlation coefficient were not included. HMO group: human milk oligosaccharide group, BF: breastfeeding, GA: gestational age, BMI_2mpp: body mass index at two months postpartum, PrePregBMI: body mass index before pregnancy.


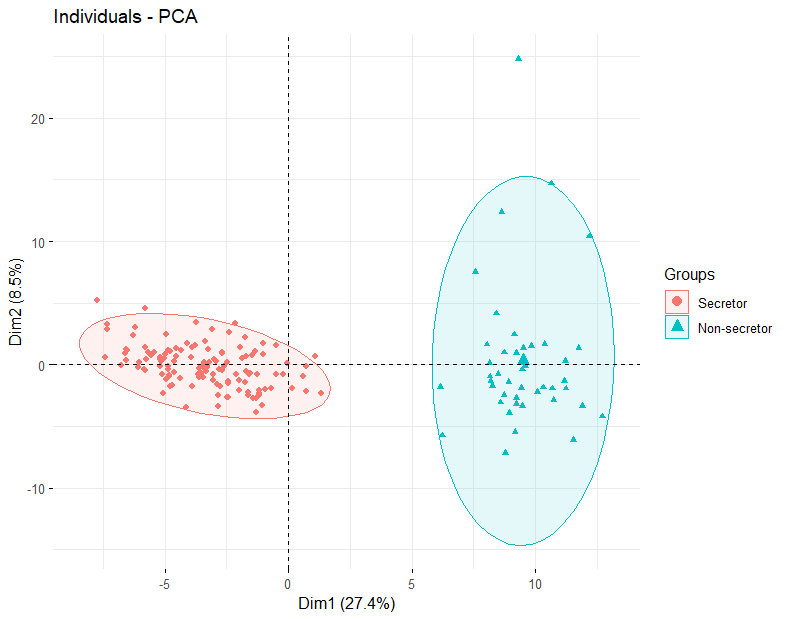


**Supplemental figure 2**. Principal component analysis (*factoextra*) of %TPH of all 135 detected peaks. Scatters and ellipses are drawn based on predefined secretor status.

**
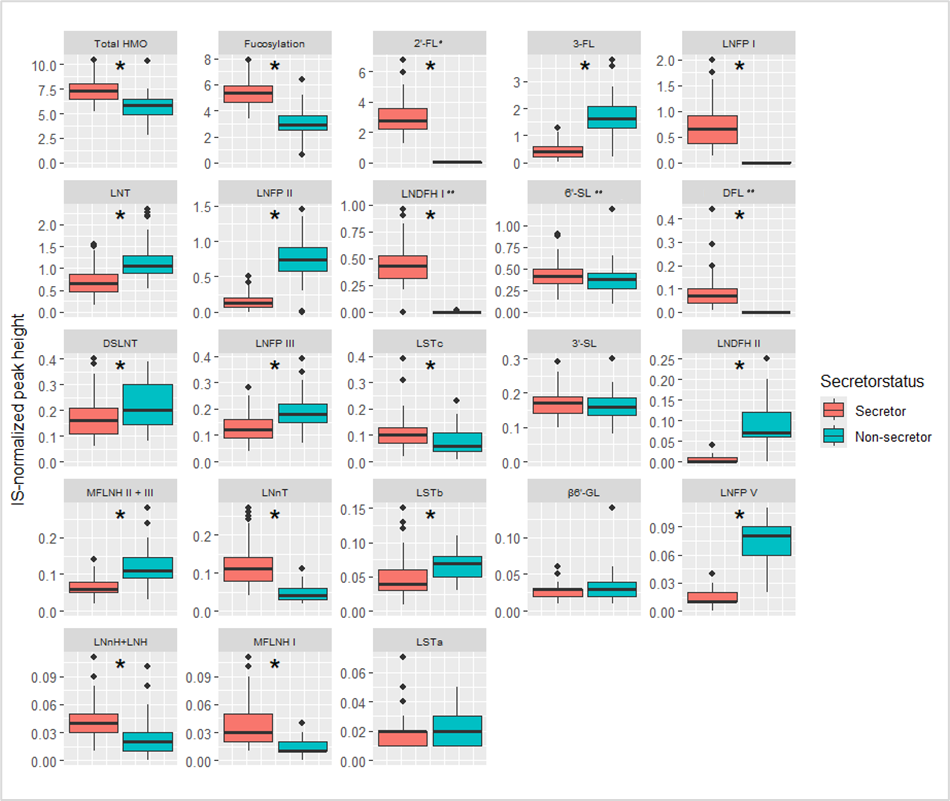
Supplemental figure 3**. Levels of major human milk oligosaccharides, fucosylation and total HMOs for non-secretors (n=43) and secretors (n=121). Differences between non-secretor and secretor were visualized (*ggplot2* in Rstudio) and tested by Wilcoxon rank sum test and adjusted for multiple testing by the false discovery rate method (*p<0.01). Boxes display median (black line) and interquartile range (IQR), with the whiskers extending to 1.5*IQR. Black dots beyond whiskers indicate outliers. Detected HMOs were normalized by dividing peak height of the annotated HMO by the peak height of the internal standard (IS) in the same sample. The peaks identified as 2’-fucosyllactose (2’-FL), 3-fucosyllactose (3-FL), lacto-N-fucopentaose (LNFP) I, II, III, and V, lacto-N-tetraose (LNT), lacto-N-difucohexaose (LNDFH) I and II, 6’-sialyllactose (6’-SL), difucosyllactose (DFL), disialyllacto-N-tetraose (DSLNT), lacto-N- sialyllacto-N-tetraose (LST) a, b and c, 3’-siallyllactose (3’-SL), monofucosyllacto-N-hexaose (MFLNH) I and II, lacto-N-neotetraose (LNnT), β1-6-galactosyllactose (β6GL), lacto-N-neohexaose (LNnH) and lacto-N-hexaose (LNH) are depicted. # = contains also traces of β4-GL. ## = contains also traces of unknown HMOs. Fucosylation is calculated as the sum of IS-normalized peak height multiplied by number of fucose-groups in the HMOs.


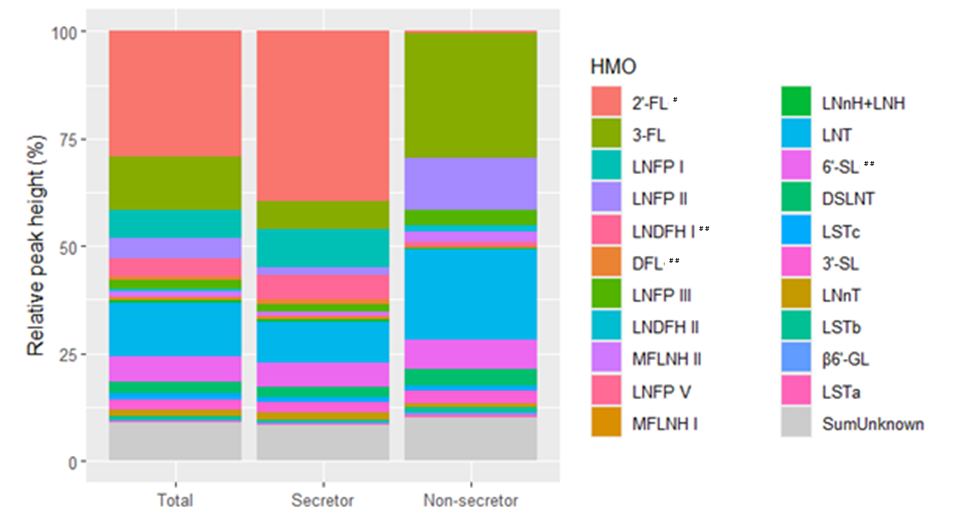


**Supplemental figure 4**. Proportion of human milk oligosaccharides separately for non-secretors (n=43), secretors (n=121) and for total population (n=164). Mean of %peak height of total peak height per sample was presented for individual annotated HMOs and non-annotated HMOs combined (*ggplot2* in Rstudio). Detected HMO peaks were normalized by dividing peak height of the annotated HMO by the peak height of the internal standard (IS) in the same sample. The peaks identified as 2’-fucosyllactose (2’-FL), 3-fucosyllactose (3-FL), lacto-N-fucopentaose (LNFP) I, II, III, and V, lacto-N-tetraose (LNT), lacto-N-difucohexaose (LNDFH) I and II, 6’-sialyllactose (6’-SL), difucosyllactose (DFL), disialyllacto-N-tetraose (DSLNT), lacto-N- sialyllacto-N-tetraose (LST) a, b and c, 3’-siallyllactose (3’-SL), monofucosyllacto-N-hexaose (MFLNH) I and II, lacto-N-neotetraose (LNnT), β1-6-galactosyllactose (β6GL), lacto-N-neohexaose (LNnH) and lacto-N-hexaose (LNH) are depicted. # = contains also traces of β4-GL. ## = contains also traces of unknown HMOs. SumUnknown presents the sum of relative peak heights of the 114 peaks which are not annotated.


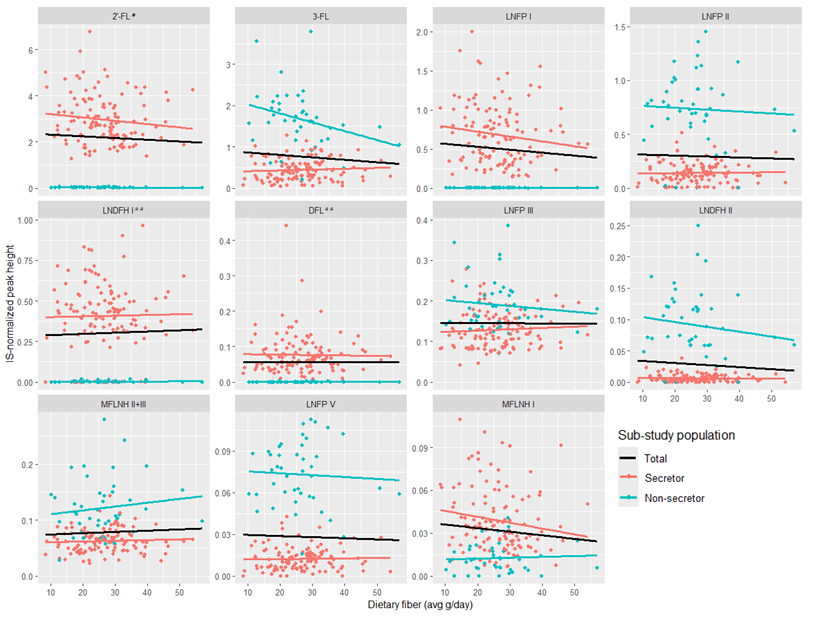


**Supplemental figure 5.** Correlation of fiber consumption of breastfeeding mothers with IS-normalized peak height of fucosylated HMOs for total sub-study population (n=164), and separately for secretors (n=121) and non-secretors (n=43). Correlation between fiber consumption and the IS-normalized peak height of each of the fucosylated HMOs is visualized (*ggplot2 in Rstudio*) and tested with Pearson correlation test (significant if p<0.05). Fiber consumption is assessed by self-reported intake in a food frequency questionnaire at two months postpartum, covering the latter four weeks, resulting in an average intake of gram/day. Human milk samples at one month postpartum are analyzed for HMOs with multiplexed capillary gel electrophoresis with laser-induced fluorescence detection. Absolute peak height of annotated fucosylated HMOs (2’-FL, 3-FL, DFL, LNDFH I and II, LNFP I, II, III, V and MFLNH I, II and III) are divided by absolute peak height of the internal standard (IS) to calculate IS-normalized peak height. # = contains also traces of β4-GL. ## = contains also traces of unknown HMOs
